# Analysis and review of techniques and tools based on machine learning and deep learning for prediction of lysine malonylation sites in protein sequences

**DOI:** 10.1093/database/baad094

**Published:** 2024-01-19

**Authors:** Shahin Ramazi, Seyed Amir Hossein Tabatabaei, Elham Khalili, Amirhossein Golshan Nia, Kiomars Motarjem

**Affiliations:** Department of Computer Science, Faculty of Mathematical Sciences, University of Guilan, Namjoo St. Postal, Rasht 41938-33697, Iran; Department of Biophysics, Faculty of Biological Sciences, Tarbiat Modares University, Jalal AleAhmad, Tehran 14117-13116, Iran; Department of Plant Sciences, Faculty of Science, Tarbiat Modares University, Jalal AleAhmad, Tehran 14117-13116, Iran; Department of Mathematics and Computer Science, Amirkabir University of Technology, No. 350, Hafez Ave, Tehran 15916-34311, Iran; Department of Statistics, Faculty of Mathematical Sciences, Tarbiat Modares University, Jalal AleAhmad, Tehran 14117-13116, Iran

## Abstract

The post-translational modifications occur as crucial molecular regulatory mechanisms utilized to regulate diverse cellular processes. Malonylation of proteins, a reversible post-translational modification of lysine/k residues, is linked to a variety of biological functions, such as cellular regulation and pathogenesis. This modification plays a crucial role in metabolic pathways, mitochondrial functions, fatty acid oxidation and other life processes. However, accurately identifying malonylation sites is crucial to understand the molecular mechanism of malonylation, and the experimental identification can be a challenging and costly task. Recently, approaches based on machine learning (ML) have been suggested to address this issue. It has been demonstrated that these procedures improve accuracy while lowering costs and time constraints. However, these approaches also have specific shortcomings, including inappropriate feature extraction out of protein sequences, high-dimensional features and inefficient underlying classifiers. As a result, there is an urgent need for effective predictors and calculation methods. In this study, we provide a comprehensive analysis and review of existing prediction models, tools and benchmark datasets for predicting malonylation sites in protein sequences followed by a comparison study. The review consists of the specifications of benchmark datasets, explanation of features and encoding methods, descriptions of the predictions approaches and their embedding ML or deep learning models and the description and comparison of the existing tools in this domain. To evaluate and compare the prediction capability of the tools, a new bunch of data has been extracted based on the most updated database and the tools have been assessed based on the extracted data. Finally, a hybrid architecture consisting of several classifiers including classical ML models and a deep learning model has been proposed to ensemble the prediction results. This approach demonstrates the better performance in comparison with all prediction tools included in this study (the source codes of the models presented in this manuscript are available in https://github.com/Malonylation).

Database URL: https://github.com/A-Golshan/Malonylation

## Introduction

### Post-translational modifications

Post-translational modifications (PTMs) are important biological regulatory mechanisms and chemical modifications in a protein after its translation (1). PTMs are often playing vital roles in protein regulation (2) and their influence on the physicochemical properties (PCPs), maturity and activity of most proteins (3–5). PTMs are adding a modified group to one or more amino acids which in turn change the chemical nature of amino acid (6). PTMs contain covalent bonds, reversible and irreversible reactions that process the events, including cutting, folding and ligand binding (7). Recent studies have shown that each modification leads to a great effect on the protein structure in addition to the functionality of the proteins (8). Moreover, PTMs are affected by activity state, localization, turnover and interactions that target proteins’ interactions with other proteins (9, 10). PTMs are involved in various molecular functionality and biological processes such as signal transduction, gene regulation, gene activation, repression, DNA repair, cell cycle control, protein–protein interactions and protein functions (11–14). Disorders in these modifications cause different diseases including cancer, diabetes and neurological diseases such as Alzheimer and Parkinson (15–18). There are more than 600 types of PTMs (19) including phosphorylation, glycosylation, methylation, ubiquitination, nitrosylation, SUMOylation, sulfation, acylation and malonylation as well as numerous others in most cellular activities (1, 6).

### Malonylation

**Figure 1.**
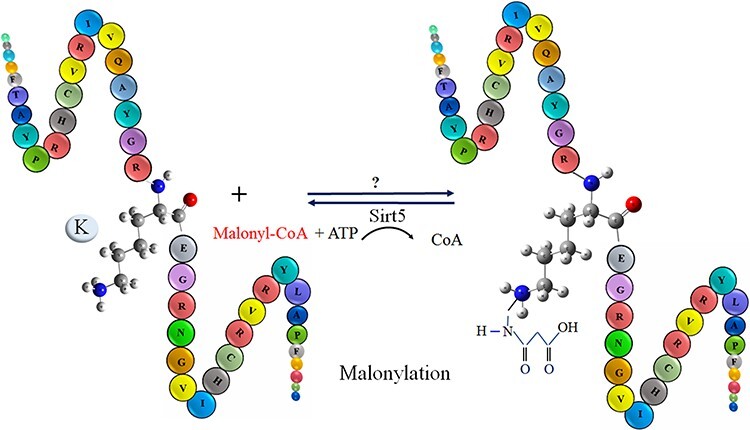
The schematic representation of malonylation process in a protein.

Malonylation was firstly observed in mammalian cells and bacterial cells via a high-throughput proteomic analysis by Peng in 2011 (20). Malonylation occurs reversibly at the lysine/k residue of a protein by adding a negatively charged malonyl group via malonyl-CoA in eukaryotic and prokaryotic cells (21). The biological process of lysine/K protein malonylation is schematically outlined in Figure 1. Furthermore, malonylation of lysine/k is confirmed to be a histone PTM. However, an abnormal histone modification is associated with disorders, such as cancers (21). As recent studies indicate, malonylation has been regulated in protein localization, enzymatic activity, protein stability and many other biochemical processes (22). Moreover, malonylation plays significant role in various metabolic pathways, for example, fatty acid synthesis and oxidation, Krebs cycle, amino acid degradation, mitochondrial respiration, glycolysis and modification of histones that are related to gene expression and chromosome configuration (23–25). Recently, it has been observed that sirt5, a member of the lysine/k deacetylases (KDACs), can catalyze the lysine/k demalonylation reaction in mammalian cells. Therefore, maybe both two modifications (acetylation and malonylation) available in the different cell compartments are regulated by lysine/k acetyltransferases and KDACs (22). According to the new studies, malonylation does have vital roles in signaling molecules in mammalian cells, mouse liver and bacterium accharopolyspora (26). Any disorder type in the malonylation process plays a potential role in type 2 diabetes, cancer and genetic disease (23–27). Recognition of malonylation sites in substrates is an important initial step in understanding the molecular mechanisms underpinning protein malonylation. Due to advancement of high-throughput mass spectrometry (MS) techniques, many malonylation-containing peptides have been discovered (28). However, because of the dynamic features of malonylation and the limitations of experiment methodologies, identifying the exact substrates or locations on a wide scale remains difficult.

### Motivation

In general, the problem of identifying malonylation sites has remained a challenge despite its functional significance (13). High-throughput experimental methods for the discovery of malonylation are costly and time-consuming. For the identification of malonylation sites, computational methods are more cost-effective and time-effective than experimental methods (29). As a result, machine learning (ML) approaches for solving such problems have grown in popularity (30, 31). There is a considerable amount of malonylation data available from various publicly accessible databases, which are valuable resources for extracting patterns to learn new models for malonylation prediction (13). Thus, there exists a crucial need for prediction methods and corresponding tools. Using ML approaches and experimental datasets, we will be able to select the optimal predictive models among those to identify malonylation sites in biological sequences (32). To date, there is a relatively large body of research works in the area of computational methods for predicting malonylation sites in protein sequences based on ML approaches (33). To translate the aforementioned malonylation site prediction problem into supervised ML problem, the protein code IDs, wherein the positive site data (polypeptide sequences with a target residue that has undergone malonylation) have been approved using the MS method, are downloaded from a database containing the PTM (e.g. malonylation). Additionally, the protein sequences are obtained from the UniProt database. Then, the positive and negative sites (polypeptide sequences with a target residue not affected by malonylation) from the target protein sequences are identified. The majority of the existing works are based on classical ML algorithms, wherein the standard pipeline of a pattern recognition chain based on the feature engineering is followed. The rest of the works are based on end-to-end deep learning (DL) in which the features are learned by a deep network or through a pre-trained network itself. The utilized algorithms are normally trained through labeled data extracted from several databases recognized as benchmark data. Some of the works are equipped with a tool to address the usability.

### Contribution of this work

In this manuscript, we perform a comprehensive review on all existing approaches, tools and databases extracted, designed and developed for the prediction of malonylation sites in protein sequences. The works are categorized and compared based on their core predictive models. The feature set is structured according to their types into three different subsets and each feature is fully described. Also, the main existing tools in the literature are described and the embedded prediction models are mentioned. To compare the performance of the shortlisted tools, a new dataset based on the most updated public databases is extracted which is then used for the sake of training and testing the tools based on the selected features utilized in the tools. Also, we have proposed our prediction approaches utilizing classical ML and DL models in order to possible improve the prediction capability of malonylation sites within the extracted protein sequences. The utilized feature set, however, is selected experimentally. The rest of the paper is as follows. Benchmark databases consisting of PTMs are explained in Section ‘Databases and Pre-processing’, wherein the required preprocessing procedures are described as well. The utilized features in the classification approaches as well as the existing categories are fully described in Section ‘Description of Features’. Section ‘Embedded Algorithms in Machine Learning-based Prediction Models’, describes the statistical learning models used in the malonylation sites prediction approaches from a general viewpoint followed by the presentation of evaluation functions used in the prediction models in Section ‘Model Evaluation’. Section ‘Approaches for Prediction of Lysine Malonylation Sites in Protein Sequences’, surveys the works existing in the literature to predict the malonylation sites in the protein sequences explaining the embedded classifiers as well as used features and classification reports categorized according to the main embedded models. The existing tools in literature are described and compared in Section ‘Analyzing and Comparing the Existing Tools’, based on the newly extracted dataset. The proposed prediction models in order to improve the prediction capability of the malonylation sites are mentioned in Section ‘Experimental Results and Improvement’, followed by discussion, computational and comparison results. Finally, Section ‘Conclusion and Future Work’, concludes the paper.

## Databases and preprocessing

### Description of existing databases

Nowadays, MS-based proteomics has accumulated a large volume of data for PTMs. Researchers in the field of scientific computations can use available experimental PTM data in publicly available databases for building different prediction models for example or running some experimental operations (6). To assure the quality of the data, they can use available databases to collect experimentally validated malonylation sites of proteins such as database PTM (dbPTM) (34), Protein lysine/k modification database (PLMD) (35) and compendium of protein lysine/k modification (CPLM) (36) as follows.

- **dbPTM**: The 2019-dbPTM has been considered as a general and comprehensive database. It contains various information about different types of PTM data from more than 30 public databases and contains 903 800 experimental sites in about 130 types of PTMs from different organisms. Nevertheless, in the updated version of dbPTM-2022, there are 2 777 000 PTM sites generated by extracting existing databases and manual curation of literature, wherein more than 2 235 000 are experimentally verified. More than 42 new modification types were added to this version. There have been a number of studies revealing the upstream regulators of PTM substrate sites in the past few years because of the increasing number of studies on the mechanism of PTMs.
- **CPLM**: A subgroup of protein PTMs, protein lysine/k modifications (PLMs), occurs at lysine/k residues of proteins and plays a key role in biological processes. Disorders of these modifications can lead to several diseases. CPLM is an online resource for studying experimentally observed PLMs extracted from the literature and public databases. Now, CPLM contains more than 592 600 sites for up to 29 types of PLMs on about 463 000 unique lysine/k residues of 105 673 proteins in 219 various species. In addition to the basic information and specifics on PLM sites of each protein entry, augmented annotations from 102 additional resources have been included to cover 13 aspects.
- **PLMD**: PLMD is a significant database for up to 20 PLMs at the protein lysine/k residues that play a critical role in regulating biological processes. PLMD contains more than about 284 700 modification events in about 53 500 proteins across 176 eukaryotes and prokaryotes that exist in PLMD data in CPLM 4.0 database.

### Data acquisition and preprocessing

Acquisition and preprocessing of protein sequences data for the sake of classification are performed as follows:

- **Data acquisition**: Data are extracted from an approved and published labeled dataset. The dataset must include both positive samples (polypeptide sequences with a target residue that has undergone malonylation) and negative samples (polypeptide sequences with a target residue not affected by malonylation). This inclusion of both types of samples is crucial to facilitate the training process for accurate malonylation site prediction.
- **Reducing the homology**: The cluster database at high identity with tolerance (CD-HIT) program is designed to reduce homology and to filter out similar sequences in the extracted database.
- **Removing the inconsistent subsequences**: The subsequences at the starting/ending point of the original protein sequences whose length is shorter than determined window length are removed from database.
- **Managing imbalance dataset**: To mitigate the imbalance sample sizes, the negative subset is under-sampled and a sample with the equal size as the positive set is created.

## Description of features

Feature extraction and selection is the key part in the pipeline of a classical ML or pattern recognition approach. Therefore, the feature description of the most important features is presented here as per their corresponding taxonomy.

### Sequence-based features

**1. Amino acid composition (AAC)**: The AAC refers to the distribution of amino acids in the sequence of a protein and information about the frequency of amino acids as a vector 1 in 20 for each protein (37, 38). This information is calculated for each amino acid in a protein/peptide by using the following equation wherein the numerator is the mean of all the amino acids of type *i* and the denominator contains the total number of amino acids in the different window sizes:


(1) $$Fraction\text{}of\text{}aai_i{}=\frac{nr.\;of\text{}amino\text{}acids\text{}of\text{}type\text{}i}{nr.\text{}of\text{}amino\text{}acids\text{}in\text{}a\text{}window\text{}size}\\{}\\$$

**2. Dipeptide composition (DPC)**: The protein sequences can be described by encoding the *n*-peptide compounds. *N* indicates the number of desired amino acids in the selected window. The dipeptide is created from the conjugation of two sequential amino acids. According to the 20 available amino acids, there is the possibility of the formation of 400 dipeptides. Information on the AAC and DPC features are achieved by using Equation 1 and Equation 2:


(2) $$Fraction\ of\ dee{p_i}\, = \frac{{nr.\ of\ deepi}}{{nr.of\ all\ possible\ dipeptides}}\\[1.5pt]$$

**3. Dipeptide deviation from expected mean (DDE)**: Dipeptide deviation from the anticipated mean (DDE) is proposed and developed in, which studies feature extraction based on amino acid combination to distinguish between epitopes and non-epitopes in cells (39, 40). The dipeptide combination (DC) of a protein sequence is primarily calculated for this purpose as:


(3) $$DC{{\mathrm{ }}_{m,{\mathrm{ }}n}} = {{H\left( {m,{\mathrm{ }}n} \right){\mathrm{ }}} \over {H - 1}}{\mathrm{ }}m,n{\mathrm{ }}\epsilon {\mathrm{ }}\left\{ {{\mathrm{A}},{\mathrm{ B}},{\mathrm{ C}},\ldots,{\mathrm{Y}}} \right\}\\[4pt]$$

where *H_mn_* is the number of paired *mn* amino acids and *H* is the protein sequence length. The theoretical mean (TM) and theoretical variance (TV) of a protein are then calculated as follows:


(4) $$\,TM{\ }\left( {m,{\ }n} \right){\ } = \frac{{{c_m}}}{{{c_h}}} \times \frac{{{c_n}}}{{{c_h}}}$$

(5) $$TV\left( {m,\,n} \right) = \frac{{TM\left( {m,\,n} \right)\left( {1\, - \,TM\left( {m,\,n} \right)} \right)}}{{H\, - \,1}}\\[4pt]$$

where *C_m_* and *C_n_* represent the number of codons encoding the first and second amino acids, respectively, and *C_H_* represents the total number of codons. Finally, DDE is calculated using TV, TM and DC as follows:


(6) $$DDE\left( {m,\,n} \right) = \frac{{DC\left( {m,\,n} \right)\left( {1\, - \,TM\left( {m,\,n} \right)} \right)}}{{\sqrt {TV\,\left( {m,\,n} \right)} }}$$

**4. Reduced alphabet**: Each amino acid is encoded as an eight-dimensional vector using the letters acid, basic, aromatic, amide, small hydroxyl, sulfur, aliphatic 1 and aliphatic 2. Consequently, a vector of 8 *L* is used to encode a sample of length *L* (41).

**5. Enhanced amino acid composition (EAAC)**: The EAAC (42) extracts information from protein sequences and calculates amino acid frequency. The EAAC can be calculated as follows:


(7) $$\begin{aligned}&{G\left( {m,{\mathrm{ }}n} \right) = {{H\left( {m,{\mathrm{ }}n} \right)} \over {H\left( n \right)}}} \nonumber\\ &{m{\mathrm{ }}\epsilon {\mathrm{ }}\left\{ {A,C,D,{\mathrm{ }}\ldots,{\mathrm{ }}Y{\mathrm{ }}} \right\}} \nonumber\\ &{n\epsilon \,\left\{ {{w_1},{w_2},\ldots,{w_L}} \right\}}\end{aligned}$$

where *H*(*m, n*) is the number of amino acid type *m, H_(n)_* is the length of the window *n*.

**6. Quasi-sequence order (QSO)**: Chou (43) first proposed the QSO descriptor, which counts the appearances of amino acids based on two distance matrices (i.e. the physicochemical distance matrix and chemical distance matrix). The details of description can be found in (43).

**7. Numerical representation for amino acids (NUM)**: By numerical mapping of amino acids in alphabetical order, the 20 standard amino acids are represented as 1, 2, 3,..., 20, and the dummy amino acid O is represented by number 21. NUM tries to turn sequences of amino acids into sequences of numerical values as in (31). With the core residue K omitted, each of the 25-residue segments in this case has 12 upstream and 12 downstream residues, creating a 24-dimensional vector.

**8. Bi-profile Bayes (BPB)**: A feature encoding technique called BPB was suggested by Shao *et al*. (44). A peptide’s probability vector is encoded by taking into account the data present in both positive and negative samples. If an unlabeled sample is represented by *S = S1, S2, …, Sn*, where each *S_j_, j* = 1, 2,…, *n* is an amino acid and *n* is the sequence window size, the BPB feature vector *P* is presented as follows:


(8) $$P\, = \,{\left[ {{x_1},\,\ldots,\,{x_n},\,{x_{n + 1}},\,\ldots,\,{x_{2n}}} \right]^T}$$

The posterior probabilities of each amino acid in the positive samples data set are represented by the letters *x*_1_*, x*_2_*, …, x_n_*, while the posterior probabilities of each amino acid in the negative samples are represented by the letters *x_n+_*_1_*, x_n+_*_2,_  *…, x_2n_*.

**9. Binary encoding of amino acids (BINA)**: BINA is an easy to use and powerful feature that converts protein sequences into numeric vectors based solely on the properties of the amino acid sequences. BINA represents each amino acid as a 21-dimensional binary vector encoded by one ‘1’ and 20 ‘0’ elements. For instance, alanine (‘A’) is represented as 100000000000000000000, cysteine (‘C’) is represented as 010000000000000000000, and so on, while the dummy amino acid ‘O’ is represented as 000000000000000000001.

**10. Profile encoding**: Profile encoding can determine the frequency of each residue and then construct the frequency sequence for every peptide because each peptide has 31 amino acid residues. The following formula can be used to determine each residue’s frequency: *L* is the sample length, *i* is the type of amino acid residue, and *C_i_* is the number of times that residue appears in the peptide. The conversion of a sample to a feature vector PV can then be done as follows:


(9) $$PV\, = \,\left[ {{F_i}} \right],\,\,\,\,i\, = \,1,\,2,\ldots,\,20$$

**11. EBPR**: An innovative approach termed the EBPR method, which combines the profile encoding method with the encoding based on attribute grouping (EBAG) method, performs better than any of its building block separately. A 31-dimensional peptide is broken into a new sequence with five groups in the first phase, which creates a new sequence using the EBAG method. The generative EBAG sequence is then encoded using the profile encoding approach, where each residue is given a value based on the frequency obtained using this method.

**12. LOGO**: LOGO encodes a sequence segment based on the frequency of amino acid occurrence as calculated by the two-sample LOGO program (45). According to this, the positive and negative sets are fed by LOGO, and as the result the frequency of each amino acid at each position is calculated based on the difference between two sets. Then, the abundance of 20 types of amino acids in each position is obtained. To generate a 24-dimensional feature vector for each 25-residue segment, the constructing amino acid frequency at each position (24 positions in total, as the central residue K is ignored in this coding scheme) is selected as final feature value (25).

**13. *k*-gram**: A *k*-gram is simply a pattern of *k* consecutive letters which could be amino acid symbols or nucleic acid symbols. Since there are 21 possible letters (20 native and 1 dummy amino acids) for each position, there are 21k possible basic *k*-grams for each value of *k* (46). *k*-gram is a the same as BINA described earlier.

**14. Enhanced grouped amino acid composition (EGAAC)**: The EGAAC algorithm converts character information of protein sequences into numerical vectors. It is a powerful feature extraction technique applied to the study of bioinformatics, including the viral PTM sites (47) and prediction of lysine/k malonylation sites (40). Based on the five physicochemical characteristics of amino acids, Lee *et al*. (48) classified 20 different types of amino acids into five categories including aliphatic group (g1: GAVLMI), aromatic group (g2: FYW), positive charge group (g3: KRH), negatively charged group (g4: DE) and uncharged group (g5: STCPNQ). The calculation formula is as follows:


(10) $$\begin{aligned}&{G\left( {m,{\mathrm{ }}n} \right) = {{H\left( {m,{\mathrm{ }}n} \right)} \over {H\left( n \right)}}} \nonumber\\ &{g{\mathrm{ }}\epsilon {\mathrm{ }}\left\{ {A,C,D,{\mathrm{ }}\ldots,{\mathrm{ }}Y{\mathrm{ }}} \right\}} \nonumber\\ &{n\epsilon \,\left\{ {{w_1},{w_2},\ldots,{w_L}} \right\}}\end{aligned}$$

where *H*(*g, n*) is the number of amino acids in group *g* within the window *n, H*(*n*) is the length of the window *n* (42).

**15. Position weight matrix (PWM)**: To find out how frequently each amino acid appears in the sequence; the PWM is calculated for each category (49). Each sample can be encoded as an *L*-dimensional vector due to the total length of the sample fragment.

**16. Position weight amino acid composition (PWAA)**: Shi *et al*. (50) proposed PWAA to retrieve protein sequence information in order to prevent loosing sequence information. It efficiently collects residual position data near the target position (51). Given an amino acid residue *x_i_, i* = 1, 2, …, 21, the PWAA technique is stated as follows:


(11) $${G_i} = \frac{1}{{m\left( {m + 1} \right)}}\mathop \sum \limits_{j = - m}^{j = m} {v_{ij}}(\,j + \frac{{\left| j \right|}}{m}),\,\,j = - m,\ldots,\,m$$

where *m* represents the number of upstream or downstream amino acids. If *x_i_* is *P’s j*th amino acid, then *v_ij_* = 1, else *v_ij_* = 0. The PWAA can generate 21-dimensional feature vectors for a protein sequence *P*.

### Evolutionary-derived features

**1. K-nearest neighbor (KNN) feature**: The KNN encoding generates features for a given sequence based on its similarity to *n* samples from both positive and negative sets. For two segments *S*_1_ = {*s*1(1), *s*1(2), …, *s*1(l)} and *S*2 = {*s*2 (1), *s*2(2), …, *s*2(l)}, the distance Dist (*S*_1_, *S*_2_) between *S*_1_ and *S*_2_ is defined as follows:


(12) $$Dist\,\left( {{S_1},\,{S_2}} \right) = \frac{{1 - \mathop \sum \nolimits_{i = 1}^l sim\left( {{s_1}\left( i \right),{s_2}\left( i \right)} \right)}}{l}$$

where *l* is the length of the segment and $sim\left( {{s_1}\left( i \right),{s_2}\left( i \right)} \right)$ measures the similarity between the amino acids ${s_1}\left( i \right)\,$ and ${s_2}\left( i \right)$ based on the normalized amino acid substitution matrix.

**2. Composition of *k*-spaced amino acid pairs (CKSAAP)**: The prediction of malonylation sites has been successfully accomplished using the CKSAAP as an efficient feature encoding method (29, 52). In a particular peptide, the occurrence frequencies of the *k*-spaced amino acid pairs are calculated using the CKSAAP encoding, which reveals the information about brief linear motifs in sequence fragments. A pair of amino acids that are *k*-spaced apart have two amino acids in them. For instance, the 441-dimensional feature vector that represents the CKSAAP encoding of a peptide for *k* = 1 is as follows:


(13) $$\frac{{\frac{{{N_{AxA}}}}{{{N_{Total}}}},\frac{{{N_{AxC}}}}{{{N_{Total}}}},\ldots,\frac{{{N_{XxX}}}}{{{N_{Total}}}}}}{{441}}$$

where *x* represents any one of 21 amino acids, *N*_Total_ represents the total number of *l*-spaced amino acid pairs. Here, CKSAAP with *k* = 1, 2, 3 and 4 were combined to encode training peptides as 2205-dimensional feature vectors.

**3. Position-specific scoring matrices (PSSMs**): The evolution of protein sequences is caused by changes in any residue, additions or deletions of a few residues, as well as gene duplication and fusion. The PSSM is widely used in biological sequence analysis to motives in nucleotide and amino acid sequences (53). The PSSM matrices encode information related to the evolutionary conservation of a protein. These extended changes have been occurring over time, and many of the similarities between the amino acid sequences have been eliminated. However, these proteins may contain several common characteristics such as common biological functions. There are negative and positive scores in the PSSM matrix. The negative score means that the provided amino acids are substituted less frequently in the arrangement, while the positive score indicates that the specified amino acids occur more frequently. The PSSM matrix in a protein with a sequence of length *L* is a matrix of *L* × 20 dimensions. Each row of the matrix is corresponding to an amino acid in the protein sequence and its columns correspond to the 20 amino acids in proteins. In this matrix *P_ij,_* the probability of the existence of the amino acid with the number *j* at position *i* from the protein is evaluated.

The POSSUM tool (https://possum.erc.monash.edu/server.jsp) has been utilized for extracting these matrices. POSSUM generates PSSM profiles of the submitted sequences by running the PSI-BLAST web server, wherein the non-redundant sequences set existing in the National Center for Biotechnology Information database have been used. For this sake, the E-value and the number of replications are set to 0.001 and 3, respectively (54). Based on this feature, S-FPSSM is generated to delicately extract evolutionary information (55). The Filtered PSSM (FPSSM) is created from the PSSM in a preprocessing step, where all PSSM negative entries are set to zero while other positive elements that are greater than an expected value $\delta $ (with a default value of 7) are set to $\delta $. As a result, the entries of an FPSSM are all integers between 0 and $\delta $. When combining two elements during matrix transformation, this step can aid in removing the negative elements’ influence on the positive ones. Based on the FPSSM, the entries of the resulting feature vector

S = (*S*_1_  ^(1),^  *…, S*_20_  ^(1)^,*…, S*_1_  ^(20)^,*…, S*_20_  ^(20)^) is described as follows:


(14) $$S_j^{(i)}={}\sum_{k=1}^Lf_{p_{k,j}}\times\delta_{k,j}$$

where *L* denotes the total number of rows of the FPSSM, *f_pk, I_* denotes the element in the *k*th row and *i*th column of FPSSM, *r_k_* denotes the *k*th residue in the sequence, and *a_i_* denotes the *i*th amino acid of 20 primary amino acids.

**4. Term frequency and inverse document frequency (TFIDF)**: To find the TFIDF coefficient, independent calculations for each term (TF and IDF) should be made (39). These terms are defined as follows:

**1. TF**(***t, d***): the number of amino acid *t* in a protein sequence, divided by the size of the protein, namely *d*.

**2. IDF(*t***): the logarithm of the total number of proteins (namely *|D|*) divided by the number of contents which include amino acid *t* (namely DF(*t*)). It is calculated as follows:


(15) $$IDF\left( t \right)\, = \,log\left( {\frac{{\left| D \right|}}{{DF\left( t \right)}}} \right)$$

Having calculated TF and IDF, TFIDF is calculated as:


(16) $$TF\, - \,IDF\left( {t,\,d} \right)\, = \,TF\left( {t,\,d} \right)\, \times \,IDF\left( t \right)$$

**5. Term frequency and category relevancy factor (TFCRF)**: This technique defines two components, positive relation frequency (PositiveRF) and negative relation frequency (NegativeRF) as follows (39):

**1. PositiveRF**: The ratio of the number of amino acids in a protein sequence (*c_i_*) that share the same trait (*t_k_*) to the total number of amino acids in the protein sequence is the factor in question. It is determined by:


(17) $$Positive\,RF\left( {{t_k},{c_d}_i} \right) = \frac{{\left| {D\left( {{t_k},{c_{j\,}}} \right)} \right|}}{{\left| {D\left( {{c_{j\,}}} \right)} \right|}}\,$$

**2. NegativeRF**: The ratio of the total number of amino acids in protein sequences other than *c_i_* that share the trait *t_k_* to the total number of amino acids in protein sequences other than *c_i_* is the factor in question. It is determined by:


(18) $$Negative\,RF\left( {{t_k},{c_i}} \right) = \frac{{\mathop \sum \nolimits_{m\, = 1,m \ne j}^{\left| C \right|} \left| {D\left( {{t_k},{c_{m\,}}} \right)} \right|}}{{\mathop \sum \nolimits_{m\, = 1,m \ne j}^{\left| C \right|} \left| {D\left( {{c_{m\,}}} \right)} \right|}}$$

where |*D*(*c_j_*)| is the number of amino acids in the set *D* and the protein *c_j_* that share the characteristic *t_k_*, and |*D*(*t_k_*, *c_j_*)| is the number of amino acids in the protein sequence *c_j_*. The category relevancy factor value (*cr fV alue*), taking into account the aforementioned formula, is defined as follows:


(19) $${}cr\text{}fV\text{}alue\text{}{(t_k,\text{}c_j)}\text{}=\frac{PositiveRF{(t_k,{}c_j{})}}{NegativeRF{(t_k,{}c_j{})}}{}$$

The relevance factor of each category has a direct correlation with PositiveRF and a reversible correlation with NegativeRF. Consequently, the following formula is the suggested weighting for feature *t_k_* in protein sequence *d_i_*:


(20) $$W_{ki}\text{}=\text{}log\text{}{(tf\text{}{(t_k,\text{}d_i)})}{}cr\text{}fV\text{}alue\text{}{(t_k,\text{}Cd_i)})$$

(21) $$log{\ }(tf{\ }\left( {{t_k},{\ }{d_i}} \right)\; \times \;\frac{{PositiveRF\left( {{t_k},\,{c_{di}}\,} \right)}}{{NegativeRF\left( {{t_k},\,{c_{di}}\,} \right))}})\\[4pt]$$

where the protein sequence *d_i_* belongs to the category *c_di_*. The performance of classification is improved through the use of normalization, which reduces the impact of sequence length. The weights are restricted to the range (0, 1). The final calculation of the TFCRF is as follows:


(22) $$\begin{array}{l}
{W_{ki}} = {\ }log{\ }\left( {tf{\ }\left( {{t_k},{\ }{d_i}} \right)} \right) \times \,crfV{\ }alue{\ }\left( {{t_k},{\ }{C_{di}}} \right)){\ } = \\
log{\ }(tf{\ }\left( {{t_k},{\ }{d_i}} \right) \times \frac{{Positive\ RF\left( {{t_k},\,{c_{di}}\,} \right)}}{{Negative\ RF\left( {{t_k},\,{c_{di}}\,} \right)}})
\end{array}\\[4pt]$$

### PCP-based features

**1. Pseudo amino acid composition (PseAAC)**: Chou (56) proposed PseAAC as an effective way to encode protein sequences. PseAAC preserves some information about the sequence order and incorporates some information about the PCP of amino acids, which is different from the amino acid frequencies. This has led to the application of the PseAAC in bioinformatics, like predicting different types of PTMs, protein subcellular localization and membrane protein types. A similar feature has been presented in (57), so we skip the detailed description.

**2. AAIndex**: AAIndex is an amino acid index database with various physicochemical and biological properties of amino acids (46, 58). The properties include hydrophobicity, polarity, polarizability, solvent/hydration potential, accessibility reduction ratio, net charge index of side chains, molecular weight, PK-N, PK-C, melting point, optical rotation, entropy of formation, heat capacity and absolute entropy. Features extracted from AAIndex have been shown to be discriminating in malonylation site prediction according to previous studies (25).

**3. Side chain property**: To generate this feature, amino acids are divided into six categories based on the characteristics of their R group: hydrophobic—aliphatic (Ala, Ile, Leu, Met and Val), hydrophobic—aromatic (Phe, Trp and Tyr), polar neutral (Asn, Cys, Gln, Ser and Thr), electrically charged—acidic (Asp and Glu), electrically charged—basic (Arg, His and Lys) and unique amino (Gly and Pro). The six different kinds of side chains are represented by a six-dimensional vector using one-hot coding or BINA (59).

**4. EBAG**: Previous studies have established that amino acid residues can be classified according to their numerous physical and chemical characteristics. Based on this notion, we use a feature encoding method known as EBAG, which divides 20 amino acid residues into four groups: hydrophobic, polar, acidic and basic. Although some intervals in amino acid sequences have no separate physical and chemical features, they can be used to determine whether a site can be modified or not, and we categorize them in the fifth group denoted by ‘X’: *C*1 group: hydrophobic (*A, F, G, I, L, MP, V, W*), *C*2 group: polar (*C, N, Q, S, T, Y*), *C*3 group: acidic (*D, E*), *C4* group: basic (*H, K, R*), *C4* group: intervals (X).

**5. BLOSUM62**: The amino acid substitution matrix is based on the alignment of amino acid sequences. The identity between the two peptide sequences does not exceed 62. The character information of the protein sequence is converted into a numerical vector. It has been widely used in bioinformatics, such as prokaryote lysine/k acetylation sites prediction (60) and malonylation site prediction (40). For example, by using the BLOSUM62 (60) feature extraction algorithm, the fragment sequence with a window length of 25 amino acids can be encoded as a 500-dimensional feature vector.

**6. Encoding based on grouped weight (EBGW**): EBGW groups 20 amino acids into seven groups according to their hydrophobicity and charge characteristics which has also been recently used for predicting malonylation sites (31). A 25-dimensional array *S_i_, I* = 1, 2, 3) of the same segmental element is produced for each group *H_i_, I* = 1; 2; 3). The element in the array will be set to 1 if the corresponding amino acid at that position belongs to the *H_i_* group; otherwise, it will be set to 0. Each array then will be broken up into smaller arrays (J-ones), which stand for *D(j)*. Len *(D(j))* is used to calculate this value by cutting the main *S_i_* from the first window which is determined using the following equation:


(23) $$\begin{array}{l}
\,Len\left( {D\left( j \right)} \right){\ } = {\ }int\frac{{j*L}}{J}\\
j{\ } = {\ }1,{\ }2, \ldots ,{\ }J{\ }L{\ } = {\ }length{\ }of{\ }segments
\end{array}$$

A vector with length *J* based on its subarrays should be defined for each group of *H_i_*, in which the *j*th element of *X_i_(j)* is determined using the following equation:


(24) $$Len\left( {D\left( j \right)} \right){\ } = X_i^j = \,\frac{{\,\mathop \sum \nolimits_j D\left( j \right)}}{{Len\left( {D\left( j \right)} \right)}}$$

**7. Z-scales**: It is an encoding scheme wherein each amino acid is characterized by five physicochemical descriptor variables, introduced and developed by Sandberg *et al*. in (61).

### Structure-based features

1. **Accessible surface area (ASA)**: ASA is a measurement of an amino acid’s solvent accessibility. It discloses crucial details regarding protein structure and interactions with other macromolecules. Additionally, it describes which amino acids are located near the surface and have a higher likelihood of undergoing PTMs (26, 62). Running SPIDER2 on each protein sequence yields the final ASA value.
2. **Secondary structure (SS)**: The proteins’ local 3D structure is determined by the secondary structure. It is made up of three regional parts: a coil, a strand and a helix. Each amino acid has the opportunity to construct one of these three local configurations due to the predicted secondary structure. With the help of this knowledge, it is possible to identify which amino acids are more organized and which are more likely to interact with other macromolecules (26, 62). The projected secondary structure by SPIDER2 is an *L × *3-dimension matrix, where *L* stands for the protein length and the columns are labeled as coil, strand and helix (pc, pe and ph, respectively).
3. **Half-sphere exposure (HSE)**: An indicator of an amino acid’s level of surface exposure on a protein is the HSE. It counts how many C∝ neighbors are contained within a half-sphere of a specified radius. An amino acid is more exposed the less C∝ neighbors it has. The position of the plane that separates the sphere with a center C∝ atom and radius ‘R’ is shown by the dotted line. Part of the protein’s C∝ backbone is represented by the thick black lines (63).
4. **Local backbone angles**: Proteins’ local structures can be represented by their local backbone angles. Local backbone angles provide us with continuous information about the local structure of proteins, in contrast to secondary structure, which offers us an idea about a local configuration with respect to constructing coil, strand or helix forms. In other words, information on the interaction of local amino acids is continuously provided by the backbone torsion angle. SPIDER2 generates probability values for the following four local backbone angles *θ, τ, χ* and Φ, which are thoroughly discussed in their original research (62, 64, 65).

### Embedded algorithms in ML-based prediction models

To get the most benefits from the extracted feature data and for the sake of prediction of malonylation sites, ML methods as a subfield of artificial intelligence must be applied. With such methods, we are able to develop algorithms for learning from and making prediction based on the constructed statistical models. There are different categories in ML methods wherein the supervised learning is a learning type which the algorithm is trained based on a labeled dataset. Informally speaking, a function which predicts the output label for the new data based on the collection of input-label pairs is learned. There are several methods categorized as the supervised learning methods in the form of learning probability models. The models can be primarily categorized as Bayesian networks, instance-based learning, neural networks, kernel machines and hidden-variables-based learning (66). However, the boarder lines among the categories are not too sharp. In Bayesian learning techniques, the prediction is made based on the probability of each hypothesis throughout their probabilities. The decision on the class ownership is made through maximizing a posteriori probability. In this concept, the hypothesis is quantified by its prior (a priori) probability and the likelihood of the data under each hypothesis is calculated by the conditional probability of the test sample when the training data are given (67). Naive Bayesian and linear Gaussian models are the main probability models in this category.

The second category of the supervised learning probability model is the instance-based learning. In contrast to the aforementioned model wherein the simple assumptions on the training data distribution are supposed, in the instant-based model a model can be highly complex in terms of statistical distribution. KNN and kernel models are prominent examples in this category (66).

The third category deals with neural networks. The concept of an artificial neural network (ANN) has been introduced in 1943 by Warren McCulloch Walter Pits (68), which was a trigger and initial points for the future development and architectures. A neural network architecture consists of neurons, links and weights. An architecture corresponding to a training sample each of feature size *n* consists of *n* input neurons (nodes). The constructing neurons are connected directly to each other, and they are propagating the activation function effects between the neurons based on the associated weights. Intrinsic features of a neural network borrowed from the human brain logical reasoning model like parallelism, adaptability, generalization, etc., make it as an efficient and favored tool in performing classification and regression tasks. Feed forward neural networks and feedback/recurrent neural networks (RNNs) are two main network types in the category of neural networks. With the rapid and vast usage of neural networks and conceptualized on top of the ANN, DL was introduced in 2006 (69, 70). The pipeline of a DL model is principally similar to an ML model; however, in contrast to the ML modeling, feature engineering is elaborated by automating the feature extraction in the DL model. DL techniques are divided into three main categories including discriminative networks (e.g. convolutional neural networks, multilayer perceptron, and RNN), generative networks (e.g. generative adversarial networks and auto encoders) and hybrid models (e.g. generative adversarial networks plus auto encoders). DL technology utilizes multiple layers to represent the abstractions of data to build highly non-linear computational model (69). One of the advantages of the DL models is their speed during the testing in comparison with the classical ML methods. However, the training time in an associated DL model is long due to the big number of learning parameters. An important feature associated with a DL model is the interpretability concept. In fact, as the feature extraction is automated in a DL model, interpreting the results obtained as the output of the DL model is necessary to be understandable by the humans.

The forth category of learning probability models includes kernel machines (66). The concept of kernel machines whose main representative is support vector machine (SVM) dates back to Aizerman *et al*. (71) with introducing of the kernel tricks. However, the maturity in the kernel theory has appeared about three decades later by Vapnik *et al*. (72) in 90s. The idea of kernel machines deals with the requirement to the developing of non-linear methods for classifying the data rather than linear techniques. By utilizing a positive definite kernel function corresponding with a similarity function (e.g. dot product) in the higher-dimensional feature space, we will perform linear calculation. However, our calculations are performed in the source space instead of the higher-dimensional feature space (73). In the other terms, with such a method so called trick, a linear classifier is utilized to solve non-linear classification problems. In fact, a kernel function is a mapping applied on the full data to transfer those from a non-linear separable space to a higher-dimensional but linearly separable space.

Finally, the last category of probability learning model is dealing with learning with the hidden variables. Hidden variables play a pillar role in many real problems in different domains including biology, economics, medical diagnostic systems, etc. For example, a hidden regulating mechanism can be a sign of complex biological systems (74). In another example, some predisposing factors in a medical diagnostic system cause an outcome like a disease represented by some symptoms. Such an outcome is considered as a hidden variable (66), so a hidden variable plays the role of an interface to transfer the information between the network parts (74). In fact, the lack of hidden variables increases the size of required model parameters significantly. To learn the hidden variables, an algorithm named expectation maximization is a generic solution. Hidden Markov models and Bayesian networks are the main areas of application of learning hidden variables (66, 74–76).

### Model evaluation

The classification models reviewed in this study have been evaluated by model evaluation parameters, mainly based on confusion matrix elements as true positive (TP), true negative (TN), false positive (FP) and false negative (FN) values. TP indicates the experimentally validated malonylation sites correctly predicted by the prediction method, and TN represents the non-malonylation sites correctly predicted. FP denotes the non-malonylation sites incorrectly predicted as malynolated sites, and finally, FN indicates the experimentally validated malonylation sites incorrectly predicted non-malonylation sites. The classification performance is evaluated by accuracy (ACC), sensitivity (SN), specificity (SP) and Kappa indicators. Furthermore, the receiver operating characteristic (ROC) curve has been extracted in some studies. The ROC curve is a plot representing the trade-off between the TP rate or sensitivity and the FP rate or 1 − specificity. A high area under the curve (AUC) in an ROC curve indicates high discriminating capability of the model in distinguishing between the binary classes. The definition of the aforementioned classification parameters is as follows:

**Accuracy**: It is the percentage of the correct predictions calculated as below.


(25) $${\mathrm{\,}}Accuracy{\ } = \frac{{TN{\mathrm{\,}} + {\mathrm{\,}}TP}}{{TN{\mathrm{\,}} + {\mathrm{\,}}FP{\mathrm{\,}} + {\mathrm{\,}}TP{\mathrm{\,}} + {\mathrm{\,}}FN}} \times {\mathrm{\,}}100$$

**Sensitivity (recall**): It indicates the percentage of malonylation sites that have been predicted correctly.


(26) $$Sensitivity{\ } = \,\frac{{TP\,}}{{TP\, + \,FN}} \times \,100$$

**Specificity**: It shows the percentage of non-malonylation sites that have been predicted correctly as non-malonylation.


(27) $$Specificity{\ } = \frac{{TN\,}}{{TN\, + \,FP}} \times \,100$$

**Kappa (*k*)**: Kappa or Cohen’s Kappa quantifies the agreement between two raters in the classification task. The formula for calculating Kappa is as follows:


(28) $$k{\ } = \,\frac{{2\left( {TP\, \times \,TN\, - \,FN\, \times FP} \right)}}{{\left( {TP\, + \,FP} \right)\left( {FP\, + \,TN} \right)\, + \,\left( {TP\, + \,FN} \right)\left( {FN\, + \,TN} \right)}}$$

Except for the ROC curve, precision–recall (PR) curves for the extracted results drawing and utilized in some works. The PR curve represents the trade-off between the precision and recall (TP rate).

**Precision**: It indicates the positive predictive value and is calculated as follows:


(29) $${\mathrm{\,}}Precision{\ } = \frac{{TF{\mathrm{\,}}}}{{TP{\mathrm{\,}} + {\mathrm{\,}}FP}} \times {\mathrm{\,}}100{\mathrm{\,}}$$

The main indicator of the PR curve is the AUC in which the high value represents both high measures. Also, to evaluate the balance between precision and recall, a score called F1-score is used which is defined as follows.

**F1-score**: It calculates the harmonic mean between precision and recall as follows:


(30) $$F1-score{\ } = \,\frac{{2\,*\,Precision\,*\,Recall}}{{Precision\, + \,Recall}}\\[1.2pt]$$

Finally, Mathew correlation coefficient (MCC), which is a preferred measure in comparison of F1-score and accuracy, is calculated as follows.

**MCC**: It summarizes the information of the corresponding confusion matrix.


(31) $$MCC{\ } = \,\frac{{TN.TP - \,FN.FP}}{{\sqrt {\left( {TP\, + \,FP} \right)\left( {TP\, + \,FN} \right)\left( {TN\, + \,FP} \right)\left( {TN\, + \,FN} \right)} }}$$

## Approaches for prediction of lysine malonylation sites in protein sequences

### Approaches based on the feature encoding methods and/or traditional ML models

In this subsection, approaches based on the classical ML methods utilizing different feature categories are mentioned. The approaches are shortly described based on their building blocks including feature engineering part and training classifiers as well as reported classification parameters.

As one of the earliest works appeared in the literature, Xu *et al*. (46) developed ‘Mal-Lys’ to predict malonylation sites based on protein sequences. Mal-Lys utilizes three types of features: sequence order information (k-grams), position-specific amino acid propensity (PSAAP) and PCP. These features are utilized to construct the model. The ‘minimum redundancy maximum replication’ approach is employed to identify the most significant features. The classification model has been trained using SVM algorithm, and the Mal-Lys prediction accuracy and the robustness are assessed using leave-one-out cross-validation (LOOCV), 6-, 8- and 10-fold cross-validations (CVs). The reported AUC is 0.81, whereas the AUC values for the 6-, 8- and 10-fold CV are similar. Using experimental data from the UniProt database, the proposed model has been validated. Finally, a tool has been developed on the top of this approach as one of the earliest tools.

The proposed method in [77] utilizes SVM to predict malonyolation sites in protein sequences by combining primary sequences and evolutionary feature vectors. The proposed method is called iLMS (identification of lysine malonylation sites) and uses features like pKSAAP, AAIndex, PSSM and DPC followed by information gain (IG) to reduce the dimension of the feature space. The classification parameters have been extracted in both CV (4,6,8,10-fold) and independent test setting. The best achieved accuracy is 0.807 corresponding to a 10-fold CV testing the mouse dataset.In another work, Du et al. [78], the authors utilized three categories of features including flanking primary sequences, conservation features and functional features to predict the lysine acylation, including acetylation, malonylation, succinylation, and glutarylation sites in a protein sequence. To feed the classifier, the features have been transformed into the numerical vectors through different coding methods. The SVM classifier has been trained by the extracted feature numerical vector. The best performance has been achieved in the presence of all features by AUC-ROC value of 0.93 for prediction of malonylation sites. The authors have experimented different feature grouping to estimate the effect of each feature category.

Wang *et al*. (31) have proposed an SVM-based classifier, Malo-Pred, for the prediction of Kmal sites in the proteome of three species (i.e. *Escherichia coli*, *Mus musculus* and *Homo sapiens*). The proposed model utilized five features including AAC, EBGW, BINA, KNN and PSSM. After evaluating the corresponding information gains, the most significant features were selected. Based on the extracted results, it was found that the KNN scores effectively capture the evolutionary similarity information around malonylation sites and demonstrate the best performance among all five features. The assessment has been performed through 10-fold CV and independent test in which accuracy scores of 0.85, 0.82 and 0.67 for *M. musculus*, *E. coli* and *H. sapiens* have been achieved, respectively. This work also confirms that different species have different biological processes and pathways, as well as unique sequence preferences for their enzymes, making it suitable for training with and predicting Kmal sites for individual species.

The presented work in (77) used SVM models to predict malonylation sites by using PseACC encoding. On a relatively small scale, they tested their method and achieved acceptable results. LOOCV and independent test have been conducted to assess the prediction performance of the classification models. They also used SVM, KNN and random forest (RF) classifiers for the malonylation site prediction. The LOOCV test on the training dataset reached an accuracy of 0.77, and the independent test on the testing dataset got 0.88. In addition, Taherzadeh *et al*. (26) used the mouse data and sequence-based and structure-based features to train an SVM-based predictor termed ‘SPRINT-Mal’ to predict malonylation sites in *H. sapiens, M. musculus* and *Saccharopolyspora Erythraea*, respectively. The model shows robust performance in predicting mouse malonylation sites as well. The 10-fold CV and the independent test have been used to assess the accuracy wherein the accuracy of 0.80 and 0.90 has been achieved, respectively. They evaluated the model with human data showing a comparable performance. Also, it has been observed that the best performance is achieved when all feature types including evolutionary information, PCP as well as HSE features are involved.

In a recent work presented by Zhang *et al*. (25), an ensemble based model utilizing 11 types of feature encoding methods has been designed. In the latter study, by using gain ratio and the combinations of feature sets, optimized feature sets have been shown to provide the best performance for three species. Concerning to this matter, the KNN based on evolutionary information, LOGO based on sequence information and AAIndex based on PCP properties have shown to be the top three important features resulting in the best performance. In benchmark tests across the three species, RF, SVM and light gradient boosting machine (LightGBM) models perform best, and LightGBM outperformed all other ML techniques trained on humans’ data sets. Also, it has been shown that the improved performance is achieved through optimal ensemble models in an independent test setting scenario across different dataset species.

In the work introduced in (62), *H. sapiens* and *M. musculus* species data have been used to extract structural and evolutionary-based features. A predictor tool ‘SEMal’ based on RF, rotation forest (RoF), SVM and Adaboost has been fed by the latter feature sets. In this approach, SS, ASA, PSSM and Local backbone angles have been extracted as feature sets. To assess the prediction capability of the model, a 10-fold CV technique has been followed. SEMal shows sensitivity (0.94 and 0.89) and accuracy (0.94 and 0.91) for *H. sapiens* and *M. musculus* species, respectively. It is worth mentioning that the combination of both structural and evolutionary-based features, mainly PSSM and SPD3, results in a better accuracy than the single feature-based models. In another work presented by Ghanbari Sorkhi *et al*. (39), they have combined seven different features using the Fisher’s score (F-score), to select the best combination. They have tried several classifiers wherein the extreme gradient boosting (XGBoost) algorithm outperforms the others in terms of prediction accuracy as reported (39). In this approach, EAAC, EGAAC, DDE, PKA, TFIDF, TFCRF and PSSM have been used as feature elements. According to the reported experiments on different feature sets, the feature TFCRF provides the best accuracy among other individual features when it is used as a single feature to feed the classifiers. The classifiers include XGBoost, SVM, RF and deep neural network (DNN). The XGBoost method outperforms other existing ML methods with an accuracy of 0.95 through a 5-fold CV setting.

Two computational methods have been presented in (78) named RF-MaloSite and DL-MaloSite, for predicting malonylation sites based on RF and DL models, respectively. The primary raw amino acid sequences are input data to the DL-MaloSite, while the RF-MaloSite scheme makes use of a wide range of biochemical, PCP and sequence-based features. Utilizing a 10-fold CV and an independent test set, RF-MaloSite demonstrated an accuracy of 0.71 and 0.70, respectively. According to a 10-fold CV and an independent set, DL-MaloSite results in an accuracy of 0.75 and 0.74, respectively. The presented tool augmented on this approach either has been trained on the data on window size of 29. Also, the first ranked feature in terms of importance is reported as PseAAC. In a different study, Ahmad *et al*. (79) used the feature extraction method PseAAC to create a new predictor called Mal-Light. The authors presented a bi-peptide approach and the PSSM to extract local evolutionary-based information according to the interactions of nearby amino acids in order to construct this model.

To predict malonylation sites, they then used LightGBM, a more sophisticated method. For *H. sapiens* and *M. musculus* proteins, using Mal-Light, accuracy has been reported to be 0.86 and 0.79, respectively. The importance of different features in an individual base has been plotted as well. In another work named IMKPse presented in (80), the general PseAAC as the classification features is used and the flexible neural tree as the classification model is engaged for the classification. In three species, including *E. coli*, *M. musculus* and *H. sapiens*, IMKPse achieved an accuracy of 0.95, 0.92 and 0.93 while tested on three data subsets in the test data set. In this work, a comprehensive analysis of the combination of different feature sets as well as their importance has been presented.

Li *et al*. (59) suggested a method based on RF to create prediction models for each encoding scheme in order to identify protein malonylation sites. For the construction of the train set, the authors utilized the PSSM, residue identity, side chain property, AAIndex and PCP properties features. Each model performance has been assessed using 10-fold CV. The proposed spatial feature, which obtained an accuracy of 0.60, outperforms the rest. In the presented approach in (29), a trained SVM classifier followed by principal component analysis (PCA) is used for the detection of malonylation sites. The classifier is fed by the feature vector extracted from BINA, PCP properties and composition of *k*-spaced acid pairs. In this approach, different combinations of CKSAAP features have been considered in order to get the valid CKSAAP feature values. To reduce the feature vector size, the PCA has been applied finally. The model has been trained on the labeled sites with a window length of 17 and tested on the 5-fold CV as well as independent set test settings. The achieved AUC-ROC for both settings is 0.96 and 0.90, respectively. Also, a tool has been developed based on the aforementioned model, which generates accuracy and plots the ROC as the examples of classification indicators in two different CV and independent test cases.

### Approaches based on DL

While the aforementioned approaches are based on the classical ML algorithms, in the approaches based on end-to-end DL, the raw data sequences are directly used for the training purposes. In feature-based deep networks, however, the features are used as the input data to the network. As one of the works using a holistic set of features, Chen *et al*. (81) have developed a DL-based algorithm and extracted features via a novel encoding method and PCP-based information. Using long short-term memory (LSTM) and RF classifiers, they built their classifier to predict malonylation sites. The encoding schemes tested include BLOSUM62, CKSAAP, binary, Z-scales, AAIndex, AAC and EAAC that were developed in this study. The performance of each predictor was assessed by a 10-fold CV and an independent test. Among these different encoding schemes, the EAAC encoding performed the best in the prediction of Kmal malonylation sites for 10-fold CV and the independent test, in terms of ACC, SN and SP, which are 88.13, 41.70 and 90.99 and 8887, 43.79 and 90.72, respectively. In the work presented in (33), convolutional neural networks (CNNs) based on the composition of physicochemical attributes, evolutional information, and sequential features that are able to identify mammals’ protein malonylation sites have been structured. The feature analysis shows the superiority of the PAAC feature for plant species and AAIndex for mammalian species, respectively, in terms of accuracy feeding RF and CNN classifiers. The best-achieved accuracy in both 10-fold CV and independent test cases are 0.764 and 0.866, respectively. In this approach, a tool has been introduced as well (82) called ‘Kmalo’. The developed tool assumes the input data in the form of fasta files or raw protein sequences and the output results which include probability measures as the classification indicator. The input data are protein sequences belonging to either mammalian or plant species.

In the work presented in (83), a CNN has been employed to identify the potential sites resulting in an approach called K net. The classification scheme makes use of three main features including EAAC, EBPR and EAAC. According to the results reported by the authors, K net obtained the AUC-ROC value of 0.79 and MCC value of 0.24, respectively. The results have been shown the superiority of the DL-based scheme (CNN) against traditional classifiers like RF, SVM, KNN and ANN. The utilized deep architecture consists of seven layers including two convolutional layers, a pooling layer, a convolutional layer, a pooling layer and a flatten layer followed by a fully connected layer. Also, to overcome the effects of imbalance data set, the authors have introduced a new verification method called split to equal validation wherein the output classification parameters are adjusted according to the ratio of the major class size to the minor class size. In another approach presented in (84), the authors utilized a DL architecture consisting of gated recurrent units (GRUs) and the DNN to predict the malonylation sites in the protein sequences. The architecture is called GRUD wherein the data is filtered through the GRU part, while the classification task is performed in the DNN part. The utilized architecture employs features including BE, BLOSUM62, BP, DC, EAAC, EBGW and PWAA resulting to a feature vector of size of 2336 elements. In this work, a method called NearMiss-2 (85) is used to handle the data size imbalance problem, which is a common challenge in the malonylation prediction problems. The achieved results reported by the authors introducing the GRUD architecture are ACC value 0.96, MCC value 0.93 and AUC-ROC value 0.99, respectively. Also, the authors showed that their introduced method outperforms the traditional ML models and the deep network architectures based on the DNN, LSTM and CNN. According to the analysis presented in the paper, the features encoding methods BLOSUM62 and DC show result in a higher accuracy than the other employed features like EBGW. However, the results corresponding to the combined features are significantly higher than the ones corresponding to the individual feature (84). To select the optimal features, Wang *et al*. (40) have used eight different models based on seven types of features containing EAAC, EGAAC, BINA, DDE, KNN, AAIndex and BLOSUM62. The proposed DL architecture is named DeepMal (40). The presented architecture consists of eight layers excluding the input layer. The achieved accuracy of the deep architecture is 0.9301 with the MCC value 0.9513 in the case of 10-fold CV. Similar to the major prediction models, the fusion of different features improves the accuracy results as expected. Finally, the proposed approach in (81) utilized a DL based on the LSTM to predict the malonylation sites in the protein sequences of mammalian species. The deep architecture consists of five layers including input layer, embedding layer, LSTM, fully connected layer and the single neuron output layer. The employed feature sets include EAAC, AAIndex and BINA, which have been extracted from the lysine-centered sequences of the lengths from 7 to 35. The evaluation method was 10-fold CV and the optimal length resulting the best AUC-ROC is 31. A tool has been developed by integrating the described deep architecture and a RF classifier. The best-achieved result corresponding to the integrated classifiers in terms of the AUC-ROC is 0.827. Table 1 summarizes the information described as the aforementioned works according to the following criteria: (i) utilized features (if applicable), (ii) detailed reported performance in terms of classification parameters, (iii) window size, (iv) sample size (P stands for the number of positive samples and N for the number of negative samples) and (v) utilized classifier(s).

**Table 1.** Specifications of the approaches used in prediction of malonylation sites in protein sequences

Method	Species	Sample size	Results of CV	Results of independent test	Feature type	Window size	Classifier
SEMal (62)	*H. sapiens* *M. musculus*	P ∼ 7635 N ∼ 7701	(10-fold) ACC = 0.94 SN = 0.94		SS, ASA, PSSM, local backbone angles	7	RF, RoF, SVM, AdaBoost
IMKPse (80)	*H. sapiens* *M. musculus* *E. coli*	P ∼ 8635 N ∼ 88 936	(10-fold) ACC = 0.97 SN = 0.98 SP = 0.96	ACC = 0.95 SN = 0.98 SP = 0.96	AAIndex	25	FNT
SPRINT-Mal (26)	*M. musculus* *S. erythraea*	P ∼ 3743 N ∼ 432 211	(10-fold) ACC = 0.80 SN = 0.49 SP = 0.81 MCC = 0.21 AUC = 0.71	ACC = 0.90 SN = 0.46 SN = 0.92 MCC = 0.22 MCC = 0.70	SS, ASA, HSE, intrinsically disordered region	11	SVM
iLMS (95)	Human Mice	P ∼ 5445 N ∼ 41 675	(4-, 6-, 8-, 10-fold) SP = 0.80	SP = 0.78 SN = 0.49 MCC = 0.26 AUC = 0.75	PCKSAAP, DPC, AAIndex	19	SVM
RF-MaloSite and DL-MaloSite (78)	Human Mice	P ∼ 4966 N ∼ 84 692	(10-fold) ACC = 0.75 SN = 0.82 SP = 0.69 MCC = 0.51	ACC = 0.74 SN = 0.80 SP = 0.68 MCC = 0.49	BINA, EAAC, AAIndex,PseAAC, physiochemical properties	25–33	DL, RF, SVM, KNN, NN
MaloPred (31)	*H. sapiens* *M. musculus* *E. coli*	P ∼ 1555,3041, 4039 N ∼ 7853, 27 499, 53 584	(10-fold) ACC = 0.86	ACC = 0.84 SN = 0.79 SP = 0.91 AUC = 0.87 MCC = 0.70	AAC, EBGW, BINA, KNN, PSSM	17–27	SVM
Kmal-sp (25)	*H. sapiens* *M. musculus* *E. coli*	P ∼ 8047 N ∼ 86 512	(10-fold) ACC = 0.83 SN = 0.83 SP = 0.87	ACC = 0.86 SN = 0.84 SP = 0.88	*k*-gram, QSO, NUM, BINA, LOGO, EBGW, AAIndex, KNN, PSSM, S-FPSSM	25	RF, SVM, KNN, LR, LGB
DeepMal (40)	*H. sapiens* *M. musculus* *E. coli*	P ∼ 9760 N ∼ 9760	(10-fold) ACC = 0.93 SN = 0.97 SP = 0.94	ACC = 0.96 SN = 0.84 SP = 0.87	EAAC, DDE, EGAAC, KNN, BLOSUM62	25	DL, DL-1, SVM, XGBoost, DNN, RNN
Mal-Lys (46)	*M. musculus*	P ∼ 458 N ∼ 3974	(6-, 8-, 10-fold) AUC-ROC = 0.81	–	*k*-gram, AAIndex, PSAAP	16	SVM
Mal-Light (79)	*H. sapiens* *M. musculus*	P ∼ 9403 N ∼ 23 021	(10-fold) ACC = 0.85 SN = 0.73 SP = 0.98 F1 = 0.83 MCC = 0.71	ACC = 0.86 SN = 0.84 SP = 0.95 F1 = 0.85	PSSM	21	XGB, AdaBoost, SVM, RF, LightGBM, LDA, QDA, bagging, DT, ETGB, MLP
Mal-Prec (29)	*H. sapiens*	P ∼ 3470 N ∼ 45 607	(10-fold) ACC = 0.90 SN = 0.89 SP = 0.91 F1 = 0.90 MCC = 0.83	ACC = 0.91 SN = 0.91 SP = 0.90 F1 = 0.91 MCC = 0.84	CKSAAP, AAIndex, KNN, BINA	17	SVM
Xiangl *et al*. (77)	Human	P ∼ 31 N ∼ 62	LOOCV ACC = 0.78 SN = 0.88 SP = 0.86 MCC = 0.75	ACC = 0.88 SN = 1 SP = 0.91 MCC = 0.75	CKSAAP, physicochemical property, biochemical property, AAIndex, binary representation	17	SVM, KNN, RF
Sorkhi *et al*. (39)	*H. sapiens* *M. musculus* *E. coli*	P ∼ 8444 N ∼ 8444	(10-fold) ACC = 0.97 SN = 0.98 SP = 0.97 MCC = 0.95 AUC = 0.98	–	EAAC, EGAAC, DDE, PKA, TFIDF, TFCRF, PSSM	5	XGBoost, SVM, RF, DNN
kmalo (82)	*H. sapiens* *M. musculus* *Triticum aestivum*	P ∼ 19 212 N ∼ 20 312	(10-fold) ACC = 0.76 SN = 0.65 SP = 0.66 MCC = 0.70 AUC = 0.67	ACC = 0.85 SN = 0.91 SP = 0.86 MCC = 0.48 AUC = 0.99	AAC, PseAAC, AAIndex, PSSM	15–35	RF, SVM, CNN
Malsite-Deep (84)	*H. sapiens* Mouse	P ∼ 4242 N ∼ 4242	(10-fold) ACC = 0.97 SN = 0.94 SP = 0.99 MCC = 0.94 AUC = 0.99	ACC = 0.98 SN = 0.99 SP = 0.99 MCC = 0.95 AUC = 0.99	BINA, PWAAPBP, EBGW, EACC, DPC, BLOSUM62	31	XGBoost, NB, GTB, RF, LightGBM, AdaBoost, SVM, LSTM, CNN, DNN, GRU, GRUD
Knet (83)	Human Mice	P ∼ 10 368 N ∼ 142 830	(10-fold) ACC = 0.92 SN = 0.36 SP = 1.0 MCC = 0.14 AUC = 0.74	ACC = 0.66 SN = 0.88 SP = 0.65 MCC = 0.21 AUC = 0.76	EAAC, EBPR, profile encoding	31	k net, RF, ANN, SVM, KNN
LEMP (81)	Human Mice	P ∼ 5288 N ∼ 88 636	(10-fold) ACC = 0.88 SN = 0.41 SP = 0.90 MCC = 0.24	ACC = 0.87 SN = 0.43 SP = 0.90 MCC = 0.24	ACC, EAAC, AAIndex, BINA, BLOSUM62, CKSAAP, Z-scales	15,1 9,23, 27,3 1,35	LSTM, LSTM-RF

Abbreviations: DT, decision tree; FNT, flexible neural tree; QDA, quadratic discriminant analysis; SS, secondary structure; GTB, gradient tree boosting; NB, Naive Bayes.

## Analyzing and comparing the existing tools

### Shortlisting and describing the tools

To review and analyze the existing tools predicting the malonylation sites, the tools that have been developed and/or augmented on top of the surveyed approaches introduced in the latter section are shortlisted. The selection criteria were on of the following: (i) availability of the tool interface in the framework of an application or web application, (ii) availability of the source code, (iii) clear description of the tools and embedded models and (iv) clear description of the training dataset. However, there were some tools wherein either the web-page address was not accessible or the source code was not available. In the latter cases, we have implemented the tool models as described in the corresponding manuscript. Also, we have recognized that in some implementations coded by the tool developers, there are some errors made resulted in a not so reliable classification performance. For example, in the implementation of the schemes proposed in (29, 40) in the corresponding code repositories, the developers have scaled the data before splitting it into train and test sets which cause information leakage between two sets trivially. In such cases, the models have been re-implemented by us to extract the reliable results. It is worth mentioning that the description of the embedded classification models as well as the prediction evaluation has been fully presented in Section 4. Finally, the following tools have been shortlisted:

**1. Mal-Lys** (46): The tool Mal-Lys has been implemented in JAVA and developed as a web application. The tool is accepting as input data a bunch of protein sequences in a fasta format and outputs the results in a tabular format according to the CV evaluation model. The corresponding embedded prediction model is the SVM. The tool is available in (86), however, according to the experiments conducted, and it detects only negative sites and does not show any sensitivity to the positive malonylation sites.

**2. Mal-Prec** (29): Mal-Prec has been implemented in MATLAB and has a similar input/output format as Mal-Lys (46). The embedded classification model is also the SVM; however, the dimension reduction is performed using PCA to reduce the feature dimensions.

**3. DeepMal** (40): DeepMal has been implemented using Python 3.6 and MATLAB14a and using the deep network architecture as well as XGBoost as its classification models. The tool does not have a web interface. The input data are in the form of fasta format to be used to extract and encode the feature values feeding the classifiers. The output of DeepMal tool is all classification parameters as well as the ROC curve.

**4. SEMal** (62): SEMal is presented via a web interface and is able to process both PSSM and SPD3 features as input data. The classification models used in the tool are RF, RoF, SVM and Adaboost. According to the source code repository (87), the tool generates all the classification parameters as well as ROC and PR curves. However, the tool processing server seems not responding to the submitted job through its web interface (88), and the source code has been used to reproduce the results.

**5. LEMP** (81): LEMP as another tool in the prediction of malonylation sites has been developed based on the prediction models presented in (81). The embedded prediction model in the LEMP is a DL network based on the LSTM and RF classifier. The web interface of the tool was not accessible during the manuscript preparation, and the model has been implemented for the sake of result generation and comparison.

**6. Mal-Light** (79): The presented approach as Mal-Light (79) embedding a lightGBM classifier does not have any launched web interface, and also the source code is not available, so the model has been implemented individually.

**7. Kmal-sp** (25): Kmal-sp is a web interface tool wherein several classifiers including RF, SVM, KNN, logistic regression (LR) and Light Gradient Boosting Machine (LGB) are embedded in order to identify the malonylation sites in the input fasta files. As the corresponding web server was not accessible during the manuscript preparation time, the model has been implemented accordingly.

**8. MaloPred** (31): MaloPred is another online prediction tool whose integrated model is based on the SVM. Since the web server was not accessible, the approach has been implemented to generate the comparison results as well.

**9. kmalo** (82): Finally, kmalo is an online tool, which calculates the class ownership probability of the detected malonylation sites. The tool always generates the same results based on the various input data, so the approach has been implemented independently from the tool and the corresponding results have been extracted.

### Comparison of the tools

To compare the performance of the shortlisted tools and their embedded models, we have collected experimentally approved malonylation sites of proteins from the dbPTM database (89). The procedure corresponding to the data extraction and analysis is as follows.

**1. Data extraction for training and testing**: The data have been extracted from the 2019-dbPTM and 2022-dbPTM databases. In this study, we have collected 3316 malonylation proteins with 8731 positive sites from 2019-dbPTM for the sake of training. Also, we have extracted 677 new and unique malonylation proteins with 678 positive sites of the 2022-dbPTM database for the testing purpose of our proposed model as well as existing tools for predicting malonylation sites.

**2. Homology reduction**: The CD-HIT program has been utilized in order to reduce the homology and to filter out similar sequences (90) (According to different PTMs prediction studies (6), we have removed redundant sequences with the threshold equal to or more than 40% similarity). As the result, a sample of 2107 protein sequences with 4555 positive sites for the training dataset and 639 proteins with 640 positive sites for the testing dataset has been extracted by the CD-HIT.

**3. Sample labeling**: The dataset is labeled according to the approved malonylation sites in the corresponding protein sequence. The negative sites indicate lysine/k amino acids, which do not have a connection with malonyl-CoA. In fact, negative data are a collection of K amino acids that lack malonyl groups and are considered as non-malonylation sites or negative samples.

The rest are considered as positive sites.

**4. Removing the trimmed sequences**: Several positive and negative sites have been found at the beginning and end of the sequenced proteins which are not symmetric with respect to the central lysine, so they cannot be processed as the symmetric sequences. Thus, due to the small volume of such rare samples, these sites have been deleted from the positive and negative datasets.

**5. Sample balancing**: As the negative sample size is intrinsically larger than the positive sample size, it is under-sampled to convert the sample set to a balance dataset.

**6. k-mers fragment extraction**: Using a (2*n* + 1, with n ranging from 3 to 17)-mers window size, fragmented sequences centered on changed sites with *n* left-hand and *n* right-hand embedding amino acids have been extracted from both positive and negative sequences.

All of the tools have been tested on the independent test data sets. The tools have been trained on the extracted data wherein a balance set of size 8442 has been used as the train set, while the balance test set is of size 1136 samples. The tools have been examined on 11 various window sizes (ranging from 7 to 35). For a window size of 7, for example, the target amino acids, lys = K residue, is placed on the center of the window, three amino acid residues upstream and three amino acid residues downstream. However, the parameters of the comparing tools have been set according to the recommendations (optimal classifier, optimal window size,...) specified (if any) in their corresponding manuscripts. The comparison results are described in Table 2 as follows.

**Table 2.** Comparison of the performance results of online tools for the prediction of malonylation sites

Tool	ACC	MCC	Kappa	SN	SP	F_1_	Web server/source code
Mal-Prec (29)	0.58	0.16	0.16	0.60	0.55	0.59	https://github.com/flyinsky6/Mal-Prec
DeepMal (DL) (40)	0.62	0.27	0.27	0.67	0.60	0.65	https://github.com/QUST-AIBBDRC/DeepMal/
DeepMal (XGB) (40)	0.73	0.46	0.46	0.80	0.66	0.75	https://github.com/QUST-AIBBDRC/DeepMal/
SEMal (62)	0.55	0.10	0.10	0.58	0.51	0.56	https://brl.uiu.ac.bd/SEMal/
LEMP (81)	0.74	0.50	0.48	0.85	0.63	0.77	http://www.bioinfogo.org/lemp (Not accessible)
Mal-Light (79)	0.60	0.21	0.21	0.70	0.50	0.64	https://brl.uiu.ac.bd/MalLight/
Kmal-sp (25)	0.67	0.34	0.33	0.77	0.56	0.70	http://kmalsp.erc.monash.edu/ (not accessible)
MaloPred (31)	0.71	0.42	0.42	0.84	0.60	0.72	http://bioinfo.ncu.edu.cn/MaloPred.aspx (not accessible)
kmalo (82)	0.70	0.41	0.40	0.78	0.62	0.72	http://fdblab.csie.ncu.edu.tw/kmalo/

According to Table 2, the tool integrated as LEMP outperforms the rest in terms of almost all classification parameters. Among these parameters, the ACC is slightly better than the corresponding parameter of DeepMal (DL) ranked the second. However, the MCC of LEMP does have a comparing value with the all others.

## Experimental results and improvement

The choice of the prediction models and classifiers to assess the discriminating capability of the features experimentally as well as the prediction capability of the models, the extracted data described in the latter section, have been utilized subjected to several ML and DL models. All selected models are feature-based whom the processed feature vectors are fed into except for the hybrid model wherein both features vector as well as raw sequence data are utilized. The finalized selected models are from RF, optimized gradient boosting family (XGBoost), statistical learner classifier (SVM and KNN) and DNN categories. The selection criteria are based on wide experimental results and literature review and the evolution pipeline of decision tree-based approaches:

RF is the method based on an ensemble model wherein the outputs of decision trees from the subsets of selected features are aggregated into the final model through majority vote mechanism. It has been introduced by Leo Breiman in 2001 based on the concept of bagging (bootstrap aggregating) methods. This bagging-based algorithm works for both classification and regression problems and also performs well for both categorical and numerical variables. Its accuracy, resistance against overfitting as well as its robustness with respect to various data types make it one of the most successfully used algorithms in the ML applications. Optimized gradient boosting is of type gradient-based decision tree algorithms with several implementations and extensions (25).

XGBoost nowadays is applied a lot aiming at increasing computational performance (speed) of the gradient-based boosted decision trees as well as getting the benefits from multiple learners instead of just one individual learner. The XGBoost algorithm is the fastest algorithm among the ones in gradient-based boosting algorithms XGBoost has been introduced in 2016 in (91). The precedence of the XGBoost is achieved through parallelization, tree pruning and hardware resource optimization. The XGBoost algorithm is able to apply regularization techniques (e.g. L1 and L2) to avoid overfitting through training. Also the algorithm is very powerful in training the sparse data due to the existence of missing values for example. In fact, the XGBoost handles the sparse data through its proposed tree learning algorithm by making the algorithm aware of the sparsity nature of the data (91). For this sake, the instance containing the missing value in the sparse matrix is classified into a predetermined added default choice in each tree node. The corresponding choice is learned from the non-missing data which trivially improves the efficiency of the algorithm by visiting just the non-missing elements (91). One of the main important characteristics of the XGBoosting algorithm is its capability in managing (optimizing) the memory space at the presence of big sample size data.

SVM is one of the commonly used classical ML algorithms in both classification and regression problems. The SVM constructs a decision boundary with a maximum margin for separating linear separable data or by pushing the data to a higher-dimensional space throughout kernel functions for making them separable. The wide applicability of SVM in both linear and non-linear classification/regression problems and their resistance against overfitting problem make it one of the most successful general-purposed ML algorithms.

A KNN algorithm is an ML algorithm that works also both for classification and regression problems. The algorithm finds the *k* examples which are nearest to the test example *x_i_* principally based on the majority vote of the encompassed labeled samples, so the value of *k* must be chosen as an odd number. For the sake of regression, however, the mean or median of the value of *k* will be taken into account. The distance is also quantified by any of the several metric functions.

The structures of deep networks are traced back to ANNs, which have been around for many decades gaining popularity from 1960s (92). An ANN can be considered as a non-linear model of neurons inspired from animal or human brain biological networks for example. The building blocks of an ANN are its neurons wherein the classification/regression task is performed through passing the feature data through the network architecture including input, hidden and output layers trained with the labels data. They have special advantages over other classifiers like the ability to learn the complex pattern, generalization capability and the robustness with respect to the noisy data. The number of hidden layers, the number of neurons in each layer, the choice of activation function and the learning rate are important parameters which normally affect on the performance of the network. The DNN models structured as multilayer architecture and furnished with mega parameters are equipped with rich libraries recently (93). Such libraries and the concrete basis of these networks provide a potential in solving highly complex classification and regression problems in various fields. There are many parameters involved in a DNN model including number of layers (*L*), total number of neurons (*N*), bias value (*B*), the choice of activation function, learning rate and the choice of optimizer and loss functions. Also, to avoid overfitting phenomenon, regularization techniques and dropout operations must apply.

**Figure 2.**
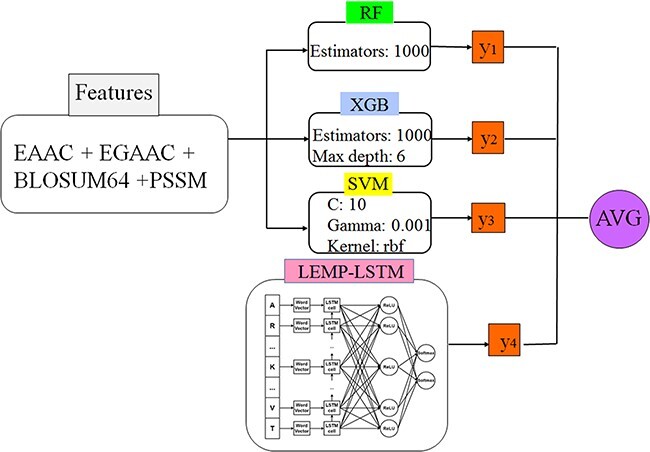
The proposed classifier as a hybrid model.

Furthermore, a hybrid model is presented to enhance the classification performance. Four classifiers as RF, SVM, XGBoost and LSTM have been integrated for this sake using an ensemble learning technique. The LSTM architecture has been taken from the LEMP tool proposed in (81) as LSTM*_WE_*. To conceptualize the mechanism, let the window size *S* be fixed. The input of the LSTM model is an amino acid sequence with window size *S*, while the concatenation of the extracted and selected feature vectors with window size *S* is the input of the other models. As can be seen in Figure 2, the first step involves using the *k*-fold CV method to extract the optimal hyperparameters of each model (classifier) followed by training. Following the training phase, the aforementioned classifiers have been integrated using the ensemble learning technique. The outputs of models have been integrated using the average voting method, wherein the weights of the models all have been considered equal. On the other hand, let *y_j_* represents the output for each model in Figure 2, evaluating the class (positive or negative) ownership probability for the tested site, then the final prediction of the hybrid model can be computed as follows:


(32) $$y = \frac{1}{4}\,\mathop \sum \limits_{i = 1}^4 {y_i}$$

It is worth to mention that in the rare case of tie, the label vote corresponding to the classifier with the higher validation accuracy will be selected. According to the assessment of the integration of single models on the test dataset, the hybrid model outperformed the other models.

The models have been trained on the features extracted from our data set as described in the latter section. The selected features include EAAC, EGAAC, BLOSUM62 and PSSM, respectively. The selection criteria are based on the reported discriminating capability in the surveyed works in the literature as well as the achieved experimental results indicating their discriminating capability. The training has been performed on a selection of fragment sizes, from 5 to 41. However, according to our computational trials, the window length of 35 is found to generate the best performance results among other lengths.

### The choice of hyperparameters in the models

The corresponding model-specific parameters have been selected according to a grid search optimization algorithm. For an RF algorithm, the number of trees has been selected from the discrete range of 100 to 1000 wherein the trees number 1000 has been finalized. The parameter ‘mtry’ has been selected as $\sqrt {nr.of\,features.} $ The parameters for training the XGBoost algorithm are optimized throughout the corresponding built-in function wherein the number of estimators and maximum depth have been set as 1000 and 6, respectively. The choice of the cost value and kernel function of the SVM classifier is from discrete range between 10^−4^ and 10^4^ (optimal value: 10 with gamma as 0.001) and radial basis function, respectively. The most significant parameter for a KNN classifier is selected from the range of 1 to 100 wherein *K* = 5 has been found to get the maximum accuracy. The DNN model uses seven-structured layers with batch normalization whose building blocks are described in Table 3.

**Table 3.** Parameters and functions of the utilized DNN model

Sequence of units in multilayers	(512, 256, 128, 128, 64, 128, 256)
Activation functions	ReLU, Softmax
Learning rate	0.001
Optimizing function	Adam
Loss function	Categorical cross entropy
Dropout	(0.5, 0.4, 0.4, 0.4, 0.4, 0.5)
Number of epochs	50
Batch size	32

**Figure 3.**
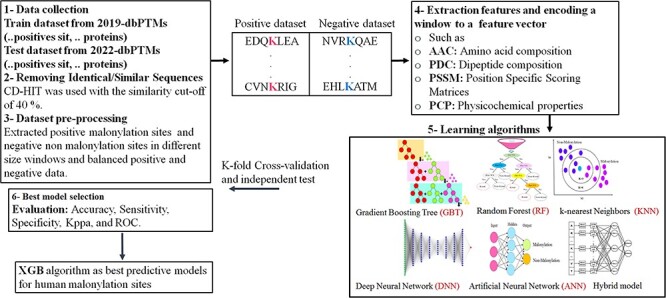
The schematic shows the ML/DL methods for prediction of malonylation sites in protein sequence: 1–3—data collection and dataset creation, 4—features extraction, 5—classifier models, 6—evaluation of the models.

Finally, the parameters of the proposed hybrid model which have been found by trial and error and also the structure of the whole model embedding the classifiers are shown in Figure 2. The performance of different trained ML models has been assessed through using the 5-fold CV test as well as an independent test set. A random sample of 10% of proteins (proteins with|lysine/k amino acids) is chosen as an independent test set, while the remainder 90% has been kept for 5-fold CV as a training/testing set (proteins with|lysine/k amino acids). Due to the small number of positive samples, 90% of all data have been used for training and CV. Also, to evaluate the performance of different models (both single method-based and ensemble models), we have assessed them on the independent test data sets. The pipeline of the overall approach is depicted in Figure 3.

### Computational results and discussion

We have trained the algorithms embedded in the aforementioned classification models with the feature categories extracted with different window sizes. The finalized selected features include EAAC, EGAAC, BLOSUM62 and PSSM according to evaluating different features combination. The analysis of the results corresponding with different window sizes indicates the optimal window size as 35. The window size of 35 (−17 to +17; with the malonylated residue in the middle) produces the most accurate prediction among other window sizes, wherein the ACC value is at its maximum value among all classification models. In terms of accuracy, all of the generated ML models are lower-bounded by 0:64 except for the KNN classifier. The sequence-based feature is the best-performing feature set, according to the results, and it improves the sensitivity and specificity of the malonylation sites prediction. To conceptualize the prediction models, we have evaluated a hybrid of numerous features in addition to comparing predictive capacities among single features. The EGAAC, which provides the best discriminating capability, has been picked as the pillar feature for the combination with other single features. Combining various features could result in a more elaborated prediction model of malonylation sites in protein sequences. We have fed the models DNN, XGBoost, SVM, RF and KNN with the full feature (EAAC + EGAAC + BLOSUM62 + PSSM) sets. The best performance of each classifier is picked and depicted in Figure 5. According to the observation, the classifier performances differ dramatically at the presence of full features comparing to a single feature set.

**Figure 4.**
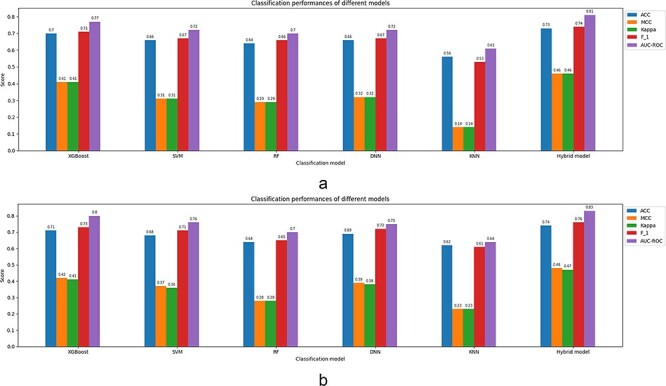
Classification parameters corresponding to the prediction models fed by the full feature set (EAAC + EGAAC + BLOSUM62 + PSSM) of window size 35 for both cases: 5-fold CV and independent test. (a) Results corresponding to a 5-fold CV. (b) Results corresponding to an independent test.

**Figure 5.**
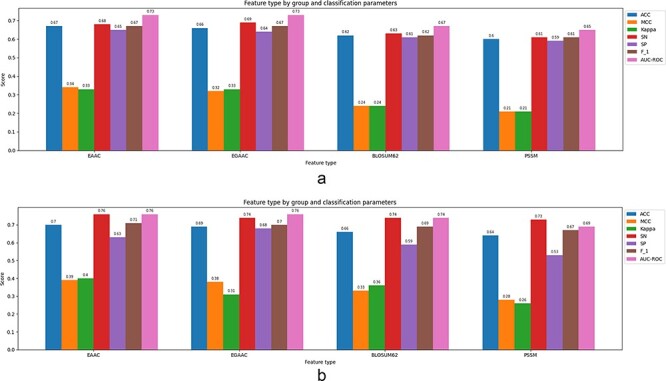
Classification parameters corresponding to the best-performing classification model (XGBoost) fed by the single feature of window size 35 for both cases: 5-fold cross-validation and independent test: (a) results corresponding to a 5-fold CV, (b) results corresponding to an independent test.

**Figure 6.**
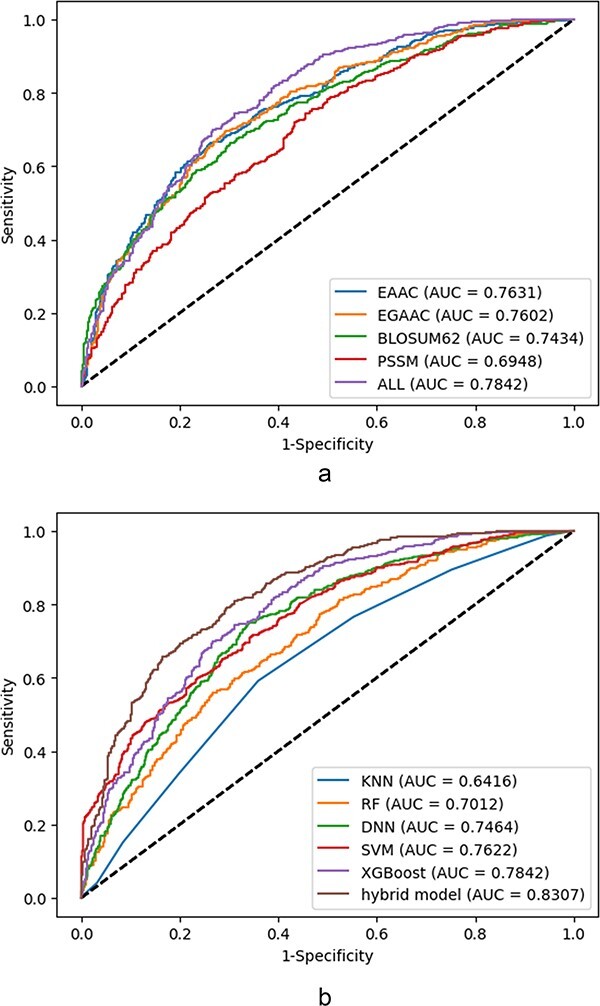
ROC curves corresponding to the individual feature and collective features: (a) ROCs corresponding to the best utilized classifier (XGBoost) based on the single feature, (b) ROCs corresponding to the utilized classifiers at the presence of all features.

Also, the XGBoost model outperforms other single ML methods for full features corresponding to a window size of 35 in a 5-fold CV setting which results in an accuracy of 0.71 and AUC-ROC of 0.80. However, the proposed hybrid model shows slightly better performance than XGBoost and achieves AUC-ROC value of 0.83 while providing the accuracy of 0.74. SVM and RF have been almost the same and have attained an accuracy and AUC-ROC of more than 0.64 and 0.70 lightly better than the DNN. The classification accuracy corresponding to the proposed hybrid model is 0.74, and the AUC-ROC is 0.83 in an independent test set evaluation outperforming the rest. The results corresponding to the single feature-based classification is presented in Figure 4 resulted from the best classifier (XGBoost) among all models excluding the hybrid scheme. According to the presented results, EAAC gives the best discriminating capability among all four selected features. It is worth mentioning that as the hybrid model is fed by both feature sets and the raw protein sequences, it has not been subjected to a single feature evaluation. The results corresponding to all features are depicted in Figure 5 in both independent test set and CV models. According to the aforementioned tables, it could be concluded that the proposed hybrid model and the XGBoost with corresponding AUC-ROC 0.80 and 0.83, respectively, outperform the other ML models for the prediction of malonylation sites in protein sequences. The achieved results through the hybrid model are also comparable with the other tools presented in Table 2. The proposed hybrid model achieves almost the same accuracy as LEMP while slightly better AUC-ROC (AUC-ROC = 0.83) than LEMP (AUC-ROC = 0.81) ranked as the most accurate tool among other shortlisted ones. Therefore, we can conclude that a window size of 35 together with hybrid model is experimentally selected as the best setting for predicting the malonylation sites. The corresponding collective ROC curves and their AUC are depicted as well in Figure 6a and b for all classification models for both all features and individual ones referring to above discussion. It is worth mentioning that all of the approaches mentioned in this manuscript including the tools re-implemented have been coded using Python and R. The main employed libraries in Python were Scikit-Learn, TensorFlow and Keras. Also, the important packages used in R were E1071, Keras, Caret and XGBoost. The training phase have been performed in real time for almost all features.

## Conclusion and future work

In this study, we have conducted a comprehensive review and presented an analysis of ML and DL-based methods for predicting lysine/K malonylation sites in protein sequences. Our aim was to provide a thorough overview of the existing approaches and their effectiveness in this specific area of research. In our study, we conducted a comprehensive survey and analysis of existing research works, manuscripts, tools and databases related to the prediction of lysine/K malonylation sites in protein sequences. We developed a taxonomy to categorize and present the important features utilized in these works. The surveyed works were categorized, explained and summarized based on the classifiers they employed, their specifications and the features they utilized. Furthermore, to facilitate a fair comparison among the developed tools, we extracted a sample data set based on the most updated databases in the field. This allowed us to evaluate and compare the performance of the different tools in a consistent manner. The tools have been trained and tested via the extracted dataset, and the classification performance has been presented.

Also, to enhance the prediction results and evaluate the discriminative power of key features in the extracted data set, various ML and DL models have been trained and tested. These models include RF, KNN, XGBoost, SVM, DNN and a hybrid model. Based on the results, the proposed hybrid model, which combines RF, SVM, XGBoost and LSTM, demonstrates superior performance compared to the other models when all selected features are present. Additionally, the introduced hybrid model outperforms the nine shortlisted tools mentioned in the manuscript. Furthermore, among the selected and studied features, the EAAC feature exhibits a stronger discriminating capability.

As the future work and in the development side, further improvement on the software development will be chased down and a tool based on the ensemble DL methods as well as simple and sophisticated prediction models will be landed online. The benefits of such a computational tool are 2-fold. At first, the properties of each feature in terms of computational complexity and discriminating capability will be explored. Secondly, the prediction and classification of the malonylation sites on the extracted protein sequences will be performed. On the research side, the elaborate prediction models will be investigated to promote the accuracy and to improve the desired classification parameters. In this approach, the models based on transfer learning and attention mechanisms as well as graph-based classification will be investigated. Finally, to elaborate feature engineering (specifically generating the new and more discriminating features) in the prediction models, utilizing proteomic data in malonylation sites or in general PTMs (e.g. (94)) which strengthen the analytic insight of the prediction models will be studied and considered.

## Data Availability

All of the source codes and datasets are available in the provided repository link https://github.com/A-Golshan/Malonylation.
